# Increased Serum Adipokines Implicate Chronic Inflammation in the Pathogenesis of Overactive Bladder Syndrome Refractory to Antimuscarinic Therapy

**DOI:** 10.1371/journal.pone.0076706

**Published:** 2013-10-01

**Authors:** Hsin-Tzu Liu, Yuan-Hong Jiang, Hann-Chorng Kuo

**Affiliations:** 1 Department of Urology, Buddhist Tzu Chi General Hospital and Tzu Chi University, Hualien, Taiwan; 2 Institute of Pharmacology and Toxicology, Tzu Chi University, Hualien, Taiwan; Imperial College London, Chelsea & Westminster Hospital, United Kingdom

## Abstract

**Objectives:**

Recent studies have shown that chronic inflammation is involved in overactive bladder (OAB) syndrome. OAB could be a subtype of neurogenic inflammation. This pilot study investigated serum adipokine levels in patients with OAB refractory to antimuscarinic therapy.

**Methods:**

Thirty consecutive patients with OAB-dry (n = 16) or OAB-wet (n = 14) refractory to previous antimuscarinic treatment were prospectively enrolled in this study, a group of 26 normal subjects without lower urinary tract symptoms served as controls. Concentrations of serum C-reactive protein (CRP), nerve growth factor (NGF), and adipokines including interleukins ([IL], IL-1β, IL-6, IL-8), tumor necrosis factor (TNF)-α, monocyte chemotactic protein (MCP)-1, insulin, and leptin were quantified using a bead-based human serum adipokine panel B kit. Data were analyzed using the LX 200 platform. Patients were further classified as having dry or wet OAB and having medical diseases or not. The serum CRP, NGF, and adipokine levels were compared between OAB patients and the controls, and between OAB subgroups.

**Results:**

The serum concentrations of CRP, NGF, IL-1β, IL-6, IL-8, and TNF-α in OAB-dry and OAB-wet patients were significantly higher than among the controls. There was no significant difference in adipokine levels between OAB-dry and OAB-wet, or between OAB patients with and without medical diseases. Serum CRP and NGF levels were significantly higher only in OAB-wet or OAB patients with medical diseases than among controls. The MCP-1 levels, on the other hand, were significantly higher in OAB-dry or OAB patients with disease, than the controls.

**Conclusions:**

Both OAB-dry and OAB-wet patients showed increased serum CRP, NGF, and adipokine levels compared with the controls, suggesting chronic inflammation of the bladder involving both peripheral and central mechanisms in all OAB patients refractory to antimuscarinic therapy. The increased serum adipokine levels were not relevant to medical diseases.

## Introduction

The clinical presentations of overactive bladder syndrome (OAB) are symptoms of urgency with or without urgency incontinence, and it is usually associated with frequency and nocturia [1]. Aging and chronic comorbidities such as hypertension, diabetes, coronary arterial disease, chronic constipation, and neurological conditions might also play roles in the pathogenesis of OAB [2], [3]. A high percentage of non-urological conditions overlap in patients with OAB, which implies possible common pathophysiology between them [4]. It is possible that some unknown circulating factors might also affect bladder dysfunction and other systemic dysfunctions.

Histological investigations of the bladder urothelium and suburothelium have shown that chronic inflammation is present in 60% of baseline biopsies of patients with OAB [5]. The inflammatory responses triggered by the activation of primary sensory neurons also induce overexpression of transient receptor potential vanilloid receptor subfamily type 1 (TRPV1) in the suburothelium as well as c-fos protein in the dorsal root ganglia, which have been demonstrated in rat models of OAB and in human bladder biopsies [6], [7].

Antimuscarinic therapy is the first line treatment for OAB [8]. Among the patients whose OAB is refractory to antimuscarinic therapy, treatment with intravesical resiniferatoxin, percutaneous tibial nerve stimulation, beta-3 adrenoceptor agonist, and botulinum toxin injection have all been reported as successful [9]. These treatments do not target the muscarinic receptors directly, but work at sensory receptors on the C-fibers, or possibly, at the central nervous system. In another study, the serum CRP level was significantly associated with storage lower urinary tract symptoms (LUTS) in women as well as residual urgency in patients with benign prostatic hyperplasia after medical treatment [10], [11].

This pilot study was conducted to investigate serum adipokine levels in a group of patients whose OAB was refractory to antimuscarinic therapy. Investigation of serum chemokine/cytokine levels could provide for the diagnosis of an OAB subtype that involves chronic inflammation; furthermore these adipokines could be potential new drug targets for treatment of OAB refractory to antimuscarinic therapy.

## Materials and Methods

This study was approved by the Institution Review Board and Research Ethics Committee of the Tzu Chi General Hospital (identifier: IRB-097-45) and written informed consent was obtained from all OAB patients and controls before their blood samples were obtained.

Thirty (17 women and 13 men) patients whose OAB was refractory to previous antimuscarinic therapy were enrolled into this study. The diagnosis of OAB was made based on the clinical symptoms of urgency or urgency incontinence and further confirmed by a three day voiding diary at enrollment. The inclusion criterion was at least three episodes of urgency/urgency incontinence within 3 days in patients diagnosed with OAB. Patients with OAB who had been treated with antimuscarinic therapy (tolterodine 4 mg daily) for more than 3 months, but in whom the urgency or urgency incontinence symptoms remained unimproved, as verified by the voiding diaries at enrollment.

Patients were further classified into OAB-wet or OAB-dry groups at enrollment based on their voiding diary records of having urgency urinary incontinence or not, respectively. Control subjects were recruited from healthy hospital employees and patients who had no urinary or systemic diseases and who were free of lower urinary tract symptoms. Medical diseases in the 3 months prior to enrollment of the OAB patients were also recorded, including diabetes, congestive heart failure, coronary arterial disease, reflux esophagitis, chronic obstructive pulmonary disease, asthma, allergic diseases, autoimmune diseases, major depression, anxiety, early dementia, minor stroke, and Parkinson's disease. Patients with BOO or urinary tract infection were not included in this study.

Five to 10 ml of blood was withdrawn and collected from each subject at enrollment. Blood samples were allowed to clot on ice for 30 to 60 min and were then centrifuged in a swinging bucket rotor at 4°C and at 3000 rpm for 15 min. Concentrations of NGF and serum adipokines including interleukin (IL) IL-1β, IL-6, IL-8, tumor necrosis factor (TNF)- α, monocyte chemotactic protein (MCP)-1, insulin, and leptin were quantified using a bead-based human serum adipokine panel B kit (Millipore, Billerica, MA, USA). Data were analyzed using the LX 200 platform (Millipore). The blood samples were assessed by one study nurse and the serum adipokines were analyzed by HTL who was blinded to the patient grouping.

Serum CRP levels were measured in a Cobas Integra 400 autoanalyzer using a particle-enhanced turbidimetric assay (Cobas Integra C-Reactive Protein Latex; Roche Diagnostics). Assays were performed at the Hualien Tzu Chi General Hospital Central Laboratory. The lower limit of detection was 0.10 mg/L.

Patients were further classified into OAB-dry or OAB-wet groups and into groups having medical diseases or not. The serum CRP, NGF, and adipokine levels were compared between patients and the controls, and between OAB subgroups. Data were expressed as means ± standard deviations. Group differences were tested using the Mann-Whitney test for non-parametric data. Differences in serum CRP and adipokine levels between control and OAB subgroups were compared. All analyses were conducted using SPSS for Windows (version 12, SPSS, Chicago, IL). A two sided p-value of less than 0.05 was taken as significant.

## Results

A total of 30 patients with OAB including 14 OAB-wet and 16 OAB-dry, and 26 control subjects were enrolled in this study. The mean age was similar between OAB-dry (61.1±9.7 years) and OAB-wet (60.9±7.8 years) but younger in the controls (32.0±8.2 years) (p = 0.000). Distribution of gender in OAB and controls were similar. (**Table 1**) The patient and control groups in this study had no overlap with previously reported patient groups.

**Table 1:** The CRP, NGF, and adipokines expressions among the controls, OAB-dry, and OAB-wet.

	(A) Control (n = 26)	(B) OAB-wet (n = 14)	(C) OAB-dry (n = 16)	(A) v (B), (A) v (C) (B) v (C)
Age	32.0±8.2 (22∼55)	60.9±7.8 (49∼73)	61.1±9.7 (39∼73)	P = 0.000, P = 0.000 P = 0.854
Gender	F:16 M:10	F:7 M:7	F:10 M:6	
NGF (pg/ml)	2.57±0.88 (1.00∼4.88)	3.66±2.45 (1.70∼11.60)	4.08±3.93 (2.20∼17.54)	P = 0.045, P = 0.028 P = 0.728
CRP (pg/ml)	0.06+0.04 (0.01∼0.16)	0.33+0.37 (0.03∼1.06)	0.17+0.23 (0.02∼0.78)	P = 0.011, P = 0.013 P = 0.238
IL-1β (pg/ml)	1.64+2.37 (0.00∼6.08)	4.68+3.10 (0.00∼10.00)	9.76+12.60 (0.00∼55.46)	P = 0.004, P = 0.000 P = 0.064
IL-6 (pg/ml)	0.79+1.05 (0.00∼3.76)	5.78+9.97 (0.65∼34.96)	2.13+2.82 (0.65∼12.11)	P = 0.000, P = 0.002 P = 0.154
IL-8 (pg/ml)	1.45+1.06 (0.00∼4.09)	4.12+3.81 (1.38∼16.58)	3.64+2.02 (1.38∼8.65)	P = 0.000, P = 0.000 P = 1.000
TNF-α (pg/ml)	0.91+0.84 (0.00∼4.64)	3.30+2.60 (0.65∼8.20)	6.04+1.51 (0.62∼23.11)	P = 0.000, P = 0.000 P = 0.608
MCP-1 (pg/ml)	104.81+37.39 (36.91∼196.08)	132.46+18.00 (48.58∼234.16)	152.08+82.34 (60.54∼401.63)	P = 0.067, P = 0.026 P = 0.580
Insulin (pg/ml)	759.8+471.7 (73.7∼2154.4)	771.58+502.54 (282.69∼1808.34)	682.60+602.84 (228.10∼2152.59)	P = 0.922, P = 0.209 P = 0.334
Leptin (pg/ml)	6242+4038 (229∼17337)	10942+14338 (472∼51014)	9960+5940 (3403∼25199)	P = 0.922, P = 0.032 P = 0.193

Data are expressed as Mean + standard deviation.

OAB: overactive bladder, IL: interleukin, TNF: tumor necrosis factor, NGF: nerve growth factor, MCP: monocyte chemotactic protein, CRP: C-reactive protein.

**Figure 1:**
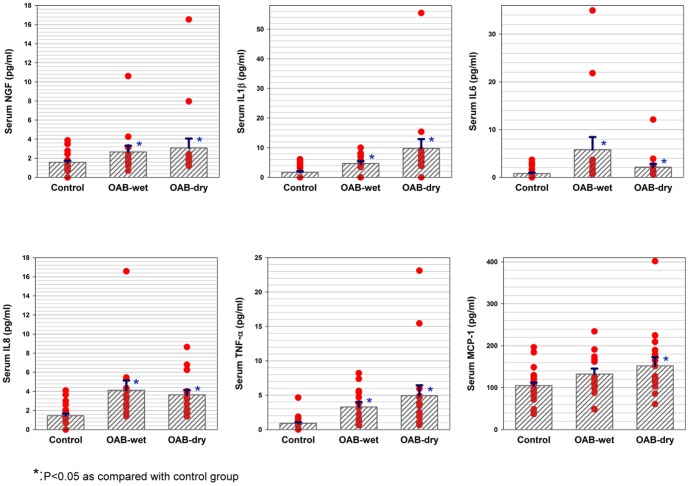
The scattered plots of serum nerve growth factor, interleukin (IL) -1β, IL-6, IL-8, tumor necrosis factor (TNF) -α, and monocyte chemotactic protein (MCP)-1 among the controls, OAB-dry and OAB-wet patients.

**Tables** **1** and **2** list the mean serum CRP, NGF, and adipokine levels for the control subjects, OAB-wet and OAB-dry groups, and the controls, OAB patients with and without medical diseases. The serum concentrations of NGF, CRP, IL-1β, IL6, IL8, and TNF-α in OAB-dry and OAB-wet patients were significantly higher than for the control subjects. (Fig. 1) There were no significant differences in the adipokine levels between OAB-dry and OAB-wet, or between OAB patients with and without medical diseases. However, serum NGF and CRP levels were significantly higher only in patients with OAB and medical diseases compared with the controls, but not in patients with OAB, but without other disease. The MCP-1 levels, on the other hand, were significantly higher only in OAB-dry or OAB with medical disease than in the control subjects.

**Table 2:** The serum NGF, CRP, and adipokines expressions among the controls and OAB patients with and without medical disease.

	(A) Control (n = 26)	(B) OAB with medical disease (n = 17)	(C) OAB without medical disease (n = 13)	(A) v (B), (A) v (C) (B) v (C)
Age	32.0+8.2 (22∼55)	60.5+9.22 (39∼73)	61.8+8.3 (49∼73)	P = 0.000, P = 0.000 P = 0.711
Gender	F:16 M:10	F:11 M:6	F:6 M:7	
NGF (pg/ml)	2.57+0.88 (1.00∼4.88)	4.65+4.21 (2.20∼17.54)	2.88+0.62 (1.70∼4.14)	P = 0.014, P = 0.093 P = 0.621
CRP (pg/ml)	0.06+0.04 (0.01∼0.16)	0.31+0.35 (0.02∼1.06)	0.11+0.63 (0.03∼0.20)	P = 0.003, P = 0.061 P = 0.419
IL-1β (pg/ml)	1.64+2.37 (0.00∼6.08)	9.09+12.47 (0.00∼55.46)	5.16+2.82 (0.00∼9.25)	P = 0.000, P = 0.001 P = 0.385
IL-6 (pg/ml)	0.79+1.05 (0.00∼3.76)	3.07+4.96 (0.65∼21.82)	4.82+9.56 (0.65∼34.96)	P = 0.000, P = 0.002 P = 0.680
IL-8 (pg/ml)	1.45+1.06 (0.00∼4.09)	4.47+3.65 (1.38∼16.58)	3.07+1.41 (1.38∼6.79)	P = 0.000, P = 0.000 P = 0.281
TNF-α (pg/ml)	0.91+0.84 (0.00∼4.64)	5.03+5.25 (0.65∼23.11)	3.03+3.92 (0.65∼15.44)	P = 0.000, P = 0.000 P = 0.065
MCP-1 (pg/ml)	104.81+37.39 (36.91∼196.08)	141.65+51.19 (60.54∼234.16)	144.59+87.80 (48.58∼401.63)	P = 0.013, P = 0.126 P = 0.483
Insulin (pg/ml)	759.8+471.7 (73.7∼2154.4)	847.2+603.6 (228.1∼2152.6)	563.1+444.4 (228.1∼1808.3)	P = 0.960, P = 0.105 P = 0.113
Leptin (pg/ml)	6242+4038 (229∼17337)	11388+11768 (841∼51015)	9150+8926 (473∼26122)	P = 0.053, P = 0.988 P = 0.300

Data are expressed as Mean + standard deviation.

## Discussion

The results of this study suggest that chronic inflammation is involved in the pathophysiology of OAB refractory to antimuscarinic therapy. Although the underlying pathophysiology for this presentation is not fully clear, identification of chronic inflammation as shown by elevated serum adipokines aids in diagnosis of this OAB subgroup and provides a new target for potential drug therapy for patients with OAB refractory to antimuscarinic therapy.

Idiopathic OAB has different etiologies including aging, BOO, bladder ischemia, and mucosal injury. Remodeling of the micturition pathways occurs following experimental BOO, denervation, spinal cord injury, cystitis, and diabetes mellitus [12]. Bladder dysfunction due to detrusor overactivity caused by aging is associated with chronic ischemia and inflammation. Compared to control subjects, however, mRNA expression levels of IL-6 in bladder tissue were significantly higher in the bladder tissues from older patients [13]. BOO causes bladder ischemia due to high intravesical pressure and increased post-void residual volumes. The oxidative stress markers and proinflammatory cytokines including IL-8-like cytokine CXCL1/CINC-1, TNF-α, and IL-6 were significantly higher in the atherosclerotic ischemic rats; these markers could be key factors in the development of bladder overactivity [14]. Put together, chronic inflammation might play a role in OAB associated with ageing, BOO and bladder injury.

Recently, some authors proposed that urothelial dysfunction, abnormal expression of sensory receptors, abnormal function of suburothelial interstitial cells, and increased excitability of detrusor muscles could be the etiologies of OAB [6], [15], [16]. Several previous studies have linked OAB to chronic inflammation of the urinary bladder. Previous studies indicate that nerve growth factor (NGF) is involved in the regulation of neural function in conditions such as spinal cord injury and denervation, as well as in inflammation and pain [17]–[19]. NGF and C-reactive protein (CRP) in urine and bladder tissue are increased in OAB patients [10], [20], [21]. The prevalence of OAB increased with increasing CRP levels in both men and women, supporting the hypothesis for the role of inflammation in the development of OAB [22]. These findings indicate that some OAB patients might have pathophysiology linked to bladder or systemic inflammatory diseases.

Neurogenic inflammation has also been linked to urinary bladder overactivity in animal models. The repetitive stimulation of C-fibers from inflammation and upregulation of sensory nerves in the bladder lead to permanent alterations or central sensitization [23]. Increased serum NGF levels have also been found in several medical and psychiatric disorders, such as asthma, allergy, Alzheimer disease, cerebrovascular accident, and physical stress [24]–[26]. Furthermore, our previous study also revealed that serum NGF levels were also increased in patients with OAB refractory to antimuscarinic therapy [27]. The elevated circulating NGF might cause an increased excitability or susceptibility of sensory receptors such as P2X3 and TRPV1 and the bladder becomes more excitable resulting in OAB [28].

Recent investigation of urinary chemokines in OAB patients also showed increases in monocyte chemotactic protein-1 (MCP-1) and some pro-inflammatory cytokines, the mean urine cytokine/chemokine levels were higher in OAB-wet than OAB-dry, suggesting a linear relationship between symptom severity and cytokine levels [29]. Increased urine cytokine levels were also noted in patients with interstitial cystitis [30]. Specific cytokines play a pivotal role for pathophysiology of OAB through upregulation of gap junction intercellular communication and connexin expressions [31]. Serum NGF levels were significantly associated with urinary NGF levels in OAB patients [27]. Taken together, increased serum and urinary NGF levels in patients with OAB refractory to antimuscarinic treatment suggest these bladder disorders could be caused by chronic inflammation.

Cytokines and chemokines play crucial roles in the pathogenesis of several chronic inflammatory diseases. In this study we chose chemokines/cytokines IL-1β, IL-6, IL-8, and TNF-α because they are associated with cystitis and systemic inflammation. IL-1β is an important mediator of the inflammatory response and is involved in a variety of cellular activities. Urinary IL-1β, IL-6, and TNF-α are significantly elevated in patients with low or high count bacterial cystitis compared to control groups [32]. Increased serum IL-6 and IL-8 in chronic cystitis could indicate an adaptive immune response after previous bladder infections. Urinary tract infections are typically accompanied by an innate immune response involving vigorous IL-6 and IL-8 production [33]. Serum pro-inflammatory cytokine (IL-1β, IL-6, TNF-α) and chemokine (IL-8) levels were significantly higher in the serum of patients with IC/BPS than in control subjects [34]. The up-regulated profile of serum IL-1β, IL-6, TNF-α, and IL-8 levels in OAB patients might potentially have a prognostic role and/or serve as a tool in choosing a proper therapeutic agent.

MCP-1 provokes mast cell activation and has chemotactic activity for monocytes that mature into macrophages at the site of inflammation [35]. Studies suggest that MCP-1 is responsible for monocyte recruitment and the chemotactic migration and activation of mast cells [36]. Levels of MCP-1 also rise during flare-up of systemic lupus erythematosus and in proteinuric states [37], [38]. Higher serum MCP-1 levels in OAB-dry, but not OAB-wet, than in the controls implies that more systemic inflammatory disorders exist among OAB-dry patients. Higher serum NGF levels in OAB-wet, but not OAB-dry, than in the controls could be explained by more neuromuscular factors in OAB-wet bladders, which might be responsible for the symptoms of urinary incontinence.

OAB and other lower urinary tract symptoms (LUTS) constitute dynamic conditions. In a longitudinal study from 1991 to 2007, there was a marked overall increase in the prevalence of urinary incontinence, the prevalence of OAB wet increased from 6% to 16% [39]. Although the increased incidence could be partially due to aging, increased medical morbidities and stress might also contribute to it. Patients with medical diseases or psychological stress have increased systemic inflammatory reactions that cause increased excitability of sensory nerves. However, the results of this study did not show significant difference of the serum adipokine levels between OAB with and without medical diseases, suggesting the increased serum cytokines originate mainly from the overactive bladder, further confirming that chronic inflammation is involved in the pathophysiology of OAB.

The main limitations of the study are the small patient number and non-parallel age between control subjects and OAB patient groups. Aging is strong risk factor in the pathogenesis development of the OAB in which that will effect on the result among the study groups, and the results also can be confounded by the pre-existing comorbidity. Inflammation is an important component of normal ageing, however, it was difficult to find control subjects who were old and free of any medical diseases in the hospital. Nevertheless, the serum adipokine levels had no significant correlation with increasing age in OAB patients (p = 0.997) or the controls (p = 0.445). Furthermore, because of the small case number of each medical disease in this study, it is not possible to compare the adipokine levels among different disease. Nevertheless, in general, we did not find a significant difference of the adipokine levels between OAB patients with and without medical diseases.

## Conclusions

In this pilot study, both OAB-dry and OAB-wet patients showed increased serum adipokine levels compared to the control subjects, suggesting chronic inflammation of the bladder involving both peripheral and central mechanisms in OAB patients refractory to antimuscarinic therapy.

## Acknowledgments

We appreciate Dr. Jen-Chine Wu, Chang Gung Medical Foundation Center for Stem cell and Translational Cancer Research, Taoyuan, Taiwan, for his technical assistance.
